# UHRF1 depletion and HDAC inhibition reactivate epigenetically silenced genes in colorectal cancer cells

**DOI:** 10.1186/s13148-019-0668-3

**Published:** 2019-05-07

**Authors:** Takeshi Niinuma, Hiroshi Kitajima, Masahiro Kai, Eiichiro Yamamoto, Akira Yorozu, Kazuya Ishiguro, Hajime Sasaki, Gota Sudo, Mutsumi Toyota, Tomo Hatahira, Reo Maruyama, Takashi Tokino, Hiroshi Nakase, Tamotsu Sugai, Hiromu Suzuki

**Affiliations:** 1 Department of Molecular Biology, Sapporo Medical University School of Medicine, S1, W17, Chuo-ku, Sapporo, 060-8556 Japan; 2 Department of Gastroenterology and Hepatology, Sapporo Medical University School of Medicine, Sapporo, Japan; 3 Project for Cancer Epigenomics, Cancer Institute, Japanese Foundation for Cancer, Tokyo, Japan; 4 Department of Medical Genome Science, Research Institute for Frontier Medicine, Sapporo Medical University School of Medicine, Sapporo, Japan; 5 Department of Molecular Diagnostic Pathology, Iwate Medical University, Morioka, Japan

**Keywords:** UHRF1, DNA methylation, Epigenetics, HDAC inhibitor, Colorectal cancer

## Abstract

**Background:**

Ubiquitin-like protein containing PHD and RING finger domains 1 (UHRF1) is a major regulator of epigenetic mechanisms and is overexpressed in various human malignancies. In this study, we examined the involvement of UHRF1 in aberrant DNA methylation and gene silencing in colorectal cancer (CRC).

**Results:**

CRC cell lines were transiently transfected with siRNAs targeting *UHRF1*, after which DNA methylation was analyzed using dot blots, bisulfite pyrosequencing, and Infinium HumanMethylation450 BeadChip assays. Gene expression was analyzed using RT-PCR and gene expression microarrays. Depletion of UHRF1 rapidly induced genome-wide DNA demethylation in CRC cells. Infinium BeadChip assays and bisulfite pyrosequencing revealed significant demethylation across entire genomic regions, including CpG islands, gene bodies, intergenic regions, and repetitive elements. Despite the substantial demethylation, however, UHRF1 depletion only minimally reversed CpG island hypermethylation-associated gene silencing. By contrast, the combination of UHRF1 depletion and histone deacetylase (HDAC) inhibition reactivated the silenced genes and strongly suppressed CRC cell proliferation. The combination of UHRF1 depletion and HDAC inhibition also induced marked changes in the gene expression profiles such that cell cycle-related genes were strikingly downregulated.

**Conclusions:**

Our results suggest that (i) maintenance of DNA methylation in CRC cells is highly dependent on UHRF1; (ii) UHRF1 depletion rapidly induces DNA demethylation, though it is insufficient to fully reactivate the silenced genes; and (iii) dual targeting of UHRF1 and HDAC may be an effective new therapeutic strategy.

**Electronic supplementary material:**

The online version of this article (10.1186/s13148-019-0668-3) contains supplementary material, which is available to authorized users.

## Introduction

Epigenetic alterations such as aberrant DNA methylation and histone modifications play essential roles in tumorigenesis [1, 2]. Cancer cells are characterized by dual DNA methylation-related changes: global hypomethylation, which can induce chromosomal instability and oncogene activation, and regional hypermethylation, which is associated with transcriptional silencing. Hypermethylation of CpG islands within gene promoter regions is a major cause of tumor suppressor gene inactivation, and a subset of cancers exhibits concurrent hypermethylation of multiple CpG islands, which is referred to as the CpG island methylator phenotype (CIMP) [3]. Because epigenetic alterations are associated with the pathogenesis and clinicopathological characteristics of cancer, they are thought to be useful biomarkers and therapeutic targets [1].

Ubiquitin-like protein containing PHD and RING finger domains 1 (UHRF1), also known as ICBP90 in human and Np95 in mouse, plays an important role in reading and maintaining the epigenetic marks [4]. UHRF1 is a multi-domain protein consisting of an N-terminal ubiquitin-like domain; a PHD domain, which interacts with methylated histones, retinoblastoma protein (pRB), and DNA methyltransferase 1 (DNMT1); a SET and RING finger-associated (SRA) domain, which interacts with hemi-methylated DNA, DNMT1, and histone deacetylase 1 (HDAC1); and a RING finger motif, which has E3 ubiquitin ligase activity [4]. UHRF1 is thought to act as a hub protein that regulates gene expression through epigenetic mechanisms, including DNA methylation and histone deacetylation, methylation, and ubiquitination [4, 5]. UHRF1 recruits DNMT1 to newly synthesized DNA to maintain DNA methylation, and genetic defects in *Uhrf1* result in significant decreases in DNA methylation in mouse embryonic stem cells [6, 7].

Recent evidence strongly suggests UHRF1 is oncogenic in human malignancies. UHRF1 is a target of E2F1 and is required for G1/S transition during the cell cycle [8, 9]. Moreover, it is overexpressed in multiple tumor types, including breast, lung, liver, pancreatic, bladder, prostate, and colorectal cancers [10–16]. Ectopic expression of UHRF1 promotes cancer cell proliferation, while UHRF1 knockdown induces cell cycle arrest, DNA damage response, and apoptosis in cancer cells [16–20]. UHRF1 is also associated with epigenetic silencing of various tumor suppressors and other tumor-related genes, including *CDKN2A*, *RB*, *BRCA1*, *RASSF1*, *PPARG*, *APC*, *CDH1*, and *RGS2* [8, 9, 15, 16, 20–24]. Inhibition of UHRF1 leads to decreased DNA methylation and/or repressive histone marks and restoration of gene expression [15, 20, 23]. Nonetheless, it is well documented that cancer cells exhibit aberrant hypermethylation of hundreds of gene promoters [25]. Thus, despite the general requirement for UHRF1 to maintain DNA methylation without bias toward specific genes [4], the involvement of UHRF1 in the epigenetic silencing of large numbers of tumor-related genes remains unclear. To address this issue, we comprehensively analyzed the effect of UHRF1 depletion on DNA methylation and gene expression in colorectal cancer (CRC) cells. We show that after UHRF1 depletion, CRC cells rapidly undergo significant DNA demethylation across the entire genome, including a number of hypermethylated CpG islands, but this only minimally restores gene expression. We also show that UHRF1 depletion plus HDAC inhibition reactivates silenced genes and suppresses CRC cell proliferation.

## Results

### UHRF1 depletion induces genome-wide DNA demethylation in CRC cells

**Fig. 1:**
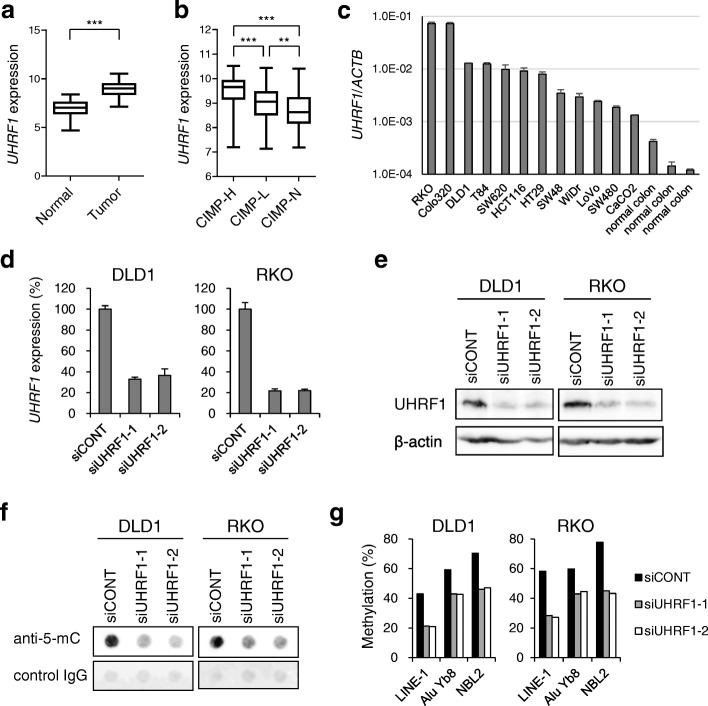
UHRF1 depletion induces global DNA demethylation in CRC cells. **a** Summaries of *UHRF1* expression in normal colon and primary CRC tumors in TCGA datasets (RSEM-normalized count). ***P < 0.001. **b** Summaries of *UHRF1* expression in CIMP-high (CIMP-H), CIMP-low (CIMP-L), and CIMP-negative (CIMP-N) CRCs in TCGA datasets. **P < 0.01, ***P < 0.001. **c** qRT-PCR analysis of *UHRF1* in CRC cell lines and normal colonic tissue. Results are normalized to *ACTB* expression. Shown are means of three replications; error bars represent SDs. **d** qRT-PCR showing *UHRF1* knockdown in CRC cells. Cells were transfected with control siRNA (siCONT) or siRNAs targeting *UHRF1* and were harvested 72 h (DLD1) or 96 h (RKO) after transfection. Results are normalized to *GAPDH* expression. Shown are means of three replications; error bars represent SDs. ***P < 0.001. **e** Western blot analysis showing UHRF1 knockdown in CRC cells. The results were confirmed in two independent experiments, and representative results are shown. **f** Dot blot analysis of 5-methylcytosine (5-mC) in CRC cells transfected with the indicated siRNAs. The results using a control IgG are shown as loading controls. The results were confirmed in two independent experiments, and representative results are shown. **g** Bisulfite pyrosequencing of repetitive elements in CRC cells transfected with the indicated siRNAs

To assess the expression of *UHRF1* in cancer, we first used RNA-seq data obtained from primary CRC and normal colonic tissues in The Cancer Genome Atlas (TCGA) study [26]. We found that *UHRF1* expression is significantly higher in CRCs than normal colon (Fig. 1a). When CRCs were categorized based on their CIMP status, both CIMP-high and CIMP-low tumors showed higher *UHRF1* expression than CIMP-negative tumors, suggesting UHRF1 may be associated with aberrant DNA methylation in CRC (Fig. 1b). In addition, quantitative RT-PCR (qRT-PCR) analysis of a series of CRC cell lines showed that all CRC cell lines expressed higher levels of *UHRF1* than normal colonic tissues (Fig. 1c).

To clarify whether UHRF1 is associated with DNA methylation in CRC cells, we performed knockdown experiments using two CIMP-high CRC cell lines (DLD1 and RKO) [27]. Transient transfection of CRC cells with two different siRNAs targeting *UHRF1* (siUHRF1-1, siUHRF1-2) successfully depleted *UHRF1* mRNA and protein (Fig. 1d, e). Dot blot analysis revealed a significant decrease in global DNA methylation levels in DLD1 cells 72 h after transfection of the siRNAs and in RKO cells 96 h after transfection (Fig. 1f). The more rapid DNA demethylation in DLD1 cells may reflect the faster cell proliferation rate than in RKO cells. We next used bisulfite pyrosequencing to assess the methylation of repetitive elements as surrogates of global DNA methylation and found reduced methylation in UHRF1-depleted cells (Fig. 1g). Depletion of UHRF1 also induced global DNA demethylation in a CIMP-negative CRC cell line (SW480) [27] and in a breast cancer cell line (MFC7), suggesting UHRF1 is required to maintain DNA methylation in multiple tumor types (Additional file 1: Figure S1). By contrast, non-cancerous HEK293 cells appeared to retain substantial levels of DNA methylation after UHRF1 depletion (Additional file 1: Figure S1).

**Fig. 2:**
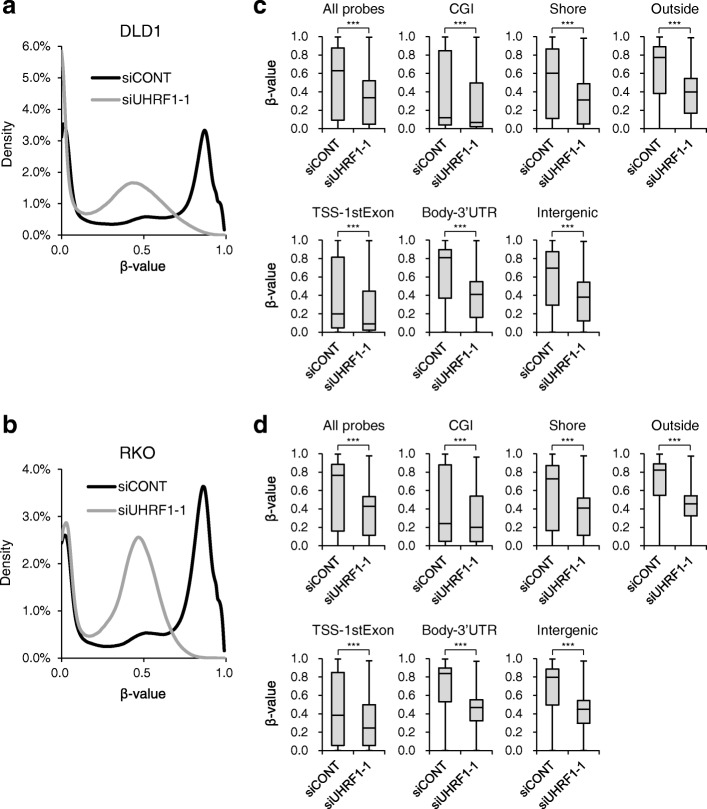
Infinium BeadChip assays revealing genome-wide DNA demethylation induced by UHRF1 depletion in CRC cells. Cells were transfected with control siRNA (siCONT) or siRNA targeting *UHRF1*, and genomic DNA was extracted 72 h (DLD1) or 96 h (RKO) after transfection. Density plots showing the *β* values of all probe sets in DLD1 (**a**) and RKO (**b**) cells transfected with the indicated siRNAs. **c** Box plots showing the *β* values of all probe sets or those located in CpG islands (CGI), CpG island shores (Shore), outside of CpG islands (Outside), transcription start sites and first exons (TSS-1stExon), gene bodies and 3′ untranslated regions (Body-3′URT), and intergenic regions (Intergenic) in DLD1 cells. ***P < 0.001. **d** Boxplots showing the *β* values of the indicated probe sets in RKO cells. ***P < 0.001

To further clarify the DNA methylation changes induced by UHRF1 depletion, we carried out Infinium HumanMethylation450 BeadChip assays with RKO and DLD1 cells. Genome-wide demethylation was clearly demonstrated in density plots of all probe sets. Peaks representing fully methylated probes (*β* value ≥ 0.8) were dramatically shifted toward intermediate methylation levels upon UHRF1 depletion (Fig. 2a, b). Box plots of all probe sets also showed significantly decreased methylation levels in cells after UHRF1 depletion (Fig. 2c, d). Categorization of the probes based on their relationship to CpG islands (CpG islands, CpG island shores, and outside of CpG islands) or gene locations (transcription start sites to the first exons, gene bodies to 3′ UTR, and intergenic regions) revealed demethylation in all genomic regions analyzed (Fig. 2c, d). In addition, we observed substantial demethylation at several CIMP marker loci (*CACNA1G*, *CDKN2A*, *CRABP1*, *IGF2*, *NEUROG1*, and *SOCS1*) in both CRC cell lines (Additional file 1: Figures S2 and S3).

### UHRF1 depletion only minimally restores expression of epigenetically silenced genes

**Fig. 3:**
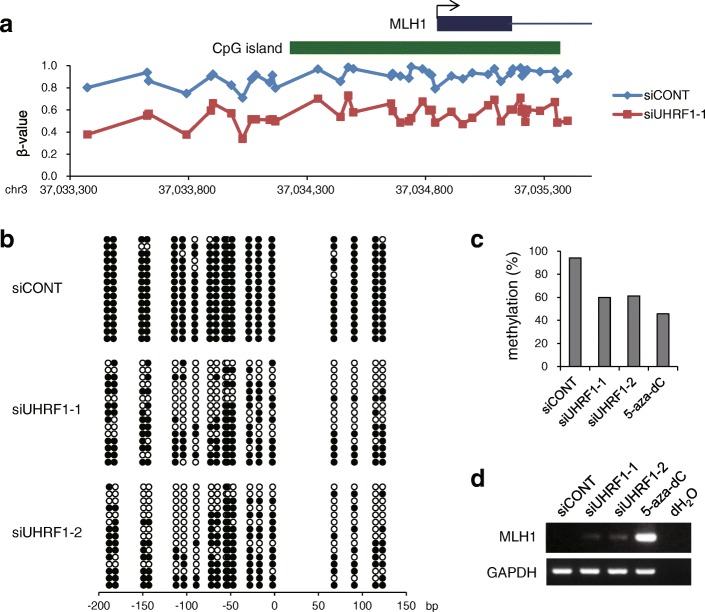
Demethylation induced by UHRF1 depletion leads to limited gene re-expression in CRC cells. **a** Results of BeadChip assays showing demethylation of *MLH1* induced by UHRF1 knockdown in RKO cells. Shown are *β* values of BeadChip probes located in the promoter region of *MLH1*. Locations of the CpG island and the first exon of *MLH1* are indicated on the top. **b** Bisulfite sequencing analysis of the *MLH1* CpG island in RKO cells transfected with the indicated siRNAs. **c** Bisulfite pyrosequencing analysis of *MLH1* in RKO cells transfected with the indicated siRNAs or those treated with 5-aza-dC. **d** RT-PCR of *MLH1* in RKO cells transfected with the indicated siRNAs or those treated with 5-aza-dC

To assess the effect of UHRF1 depletion on the methylation status of affected genetic loci, we focused on a tumor suppressor gene, *MLH1*, which is silenced in association with CpG island hypermethylation in RKO cells. BeadChip assays revealed that UHRF1 depletion leads to substantial demethylation across the entire *MLH1* CpG island (Fig. 3a). Bisulfite sequencing and bisulfite pyrosequencing confirmed that demethylation was induced by both siRNAs targeting *UHRF1* (Fig. 3b, c). We also analyzed RKO cells treated with the DNMT inhibitor 5-aza-2′-deoxycytidine (5-aza-dC) as a positive control for demethylation and re-expression (Fig. 3c, d). However, RT-PCR analysis revealed that UHRF1 depletion induced only limited re-expression of *MLH1* compared to 5-aza-dC (Fig. 3d).

### UHRF1 depletion plus HDAC inhibition reactivates epigenetically silenced genes

**Fig. 4:**
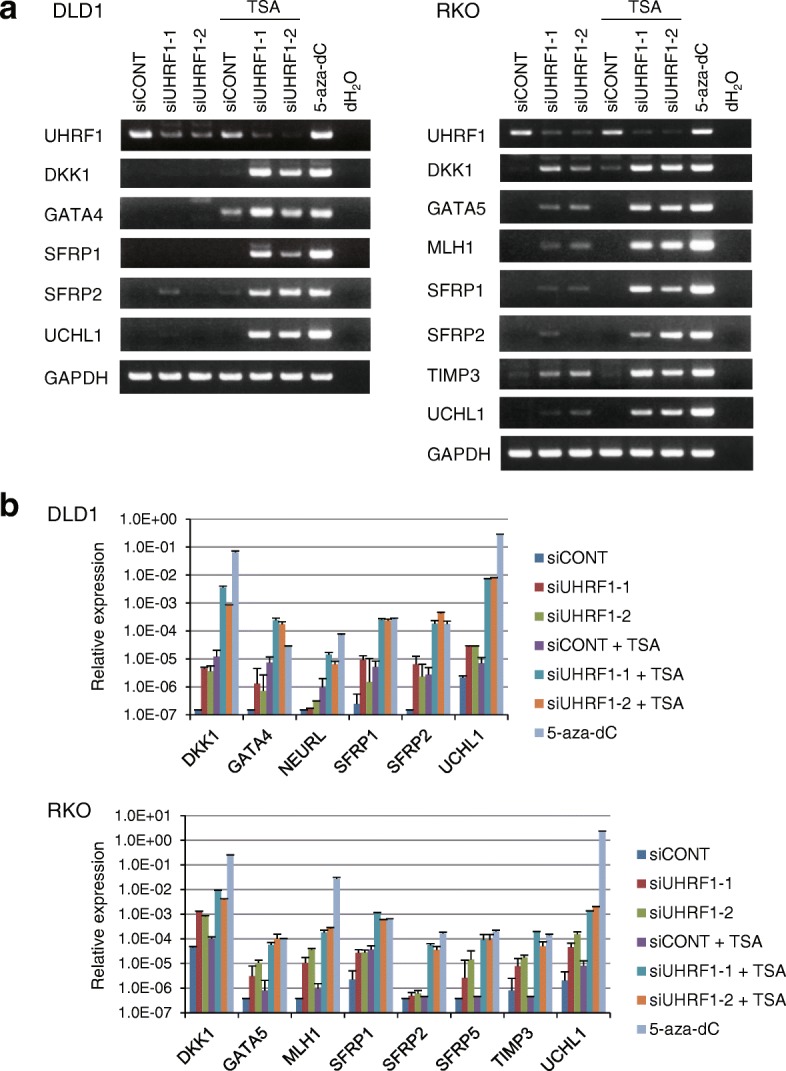
UHRF1 depletion plus HDAC inhibition restore the expression of epigenetically silenced genes in CRC cells. **a** RT-PCR analysis of *UHRF1* and epigenetically silenced genes in DLD1 (left) and RKO cells (right). Cells were transfected with the indicated siRNAs and incubated for 48 h (DLD1) or 72 h (RKO), after which the cells were treated with or without TSA for 24 h. Cells treated with 5-aza-dC are shown as positive controls for gene reactivation. **b** qRT-PCR analysis of epigenetically silenced genes in the same samples used in **a**. The results are normalized to *GAPDH* expression. Shown are means of three replications; error bars represent SDs

**Fig. 5:**
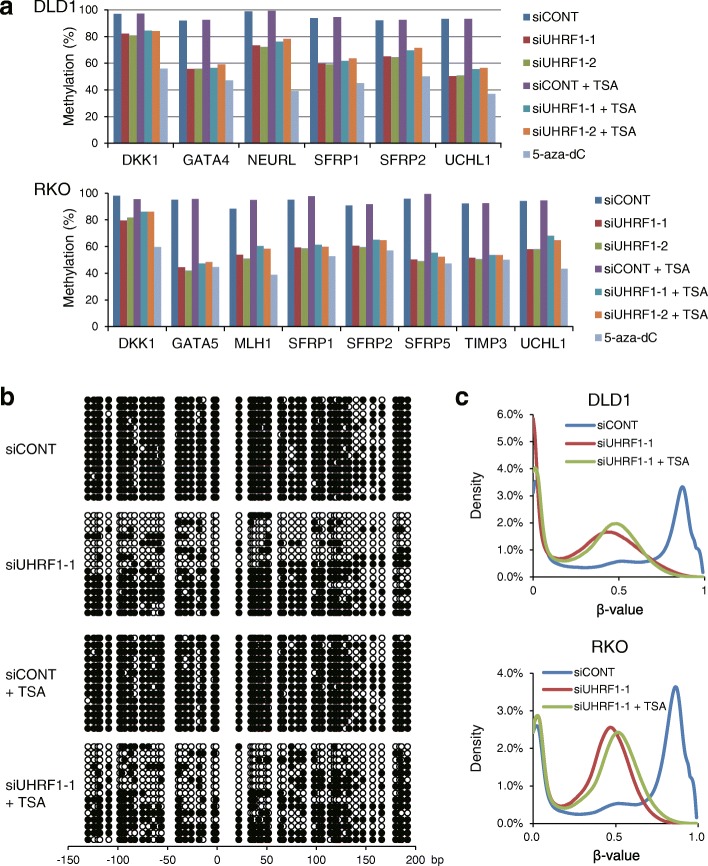
Additional HDAC inhibition in UHRF1-depleted CRC cells does not induce further DNA demethylation but does induce histone acetylation of epigenetically silenced genes. **a** Bisulfite pyrosequencing analysis of epigenetically silenced genes in DLD1 and RKO cells. Cells were transfected with the indicated siRNAs and incubated for 48 h (DLD1) or 72 h (RKO), after which they were treated with or without TSA for 24 h. Cells treated with 5-aza-dC are shown as positive controls for demethylation. **b** Bisulfite sequencing analysis of the *SFRP1* CpG island in DLD1 cells with the indicated siRNAs and treatment. Open and filled circles depict unmethylated and methylated CpG sites, respectively. Locations relative to the transcription start site are shown below. **c** Density plots of the Infinium BeadChip assay results in DLD1 and RKO cells with the indicated siRNAs and treatment

The results summarized above suggest demethylation induced by UHRF1 depletion is not sufficient to fully open the chromatin structures of epigenetically silenced genes in cancer cells. To test this hypothesis, we treated UHRF1-depleted CRC cells with trichostatin A (TSA), a HDAC inhibitor, and assessed the expression of well-characterized tumor suppressor genes silenced by CpG island hypermethylation. We found that UHRF1 depletion alone restored gene expression to a relatively limited degree, whereas the combination of UHRF1 depletion plus TSA restored the gene expression in both DLD1 and RKO cells (Fig. 4a, b; Additional file 1: Figure S4). Similar results were also observed in SW480 and MCF7 cells (Additional file 1: Figure S5). Bisulfite pyrosequencing and bisulfite sequencing confirmed that, by itself, UHRF1 depletion decreased methylation levels in affected CpG islands, and TSA did not induce further demethylation (Fig. 5a, b; Additional file 1: Figure S6). Moreover, BeadChip assays and bisulfite pyrosequencing of repetitive elements confirmed that TSA treatment did not significantly affect global DNA methylation levels after UHRF1 depletion (Fig. 5c, Additional file 1: Figure S6), and similar results were observed in SW480 and MCF7 cells (Additional file 1: Figure S7).

To further clarify the mechanism underlying gene reactivation by UHRF1 depletion plus TSA, we assessed histone acetylation in RKO cells. We found that the levels of acetylated histone H3 lysine 9 (H3K9ac) at the promoters of tumor-related genes remained low despite UHRF1 depletion, but they were increased by UHRF1 depletion plus TSA (Additional file 1: Figure S8). These results suggest that targeting UHRF1 and HDAC is effective for reactivating silenced genes in cancer cells.

### UHRF1 depletion plus HDAC inhibition strongly suppresses CRC cell proliferation

**Fig. 6:**
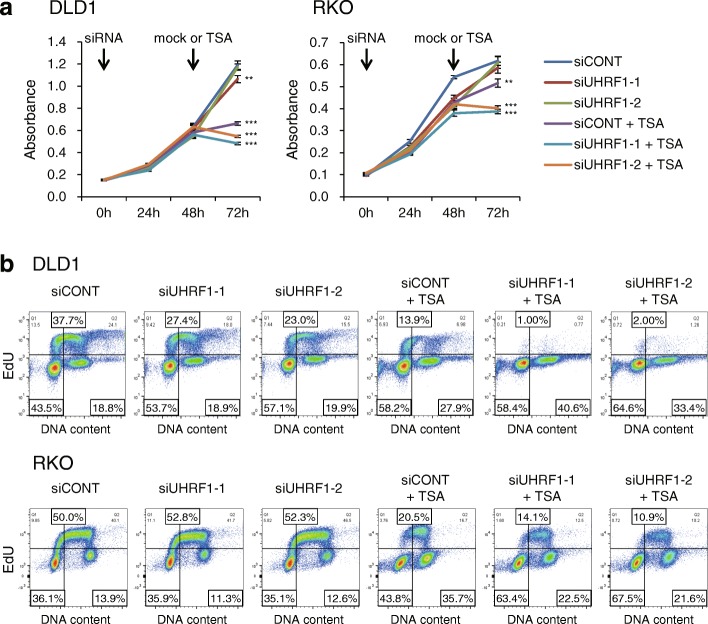
UHRF1 depletion plus HDAC inhibition suppresses CRC cell proliferation. **a** Cell viability assays in DLD1 (left) and RKO (right) cells. Cells were transfected with the indicated siRNAs and incubated for 48 h, after which they were incubated with or without TSA for 24 h. Cell viabilities were assessed at the indicated time points. Shown are means of eight replications; error bars represent SDs. **P < 0.01, ***P < 0.001. **b** EdU cell proliferation assays in DLD1 (upper) and RKO (lower) cells treated with the indicated siRNAs alone or plus TSA. The results were confirmed in at least three independent experiments, and representative results are shown (also see Additional file 1: Figure S5).

The reactivation of multiple tumor-related genes by combined UHRF1 depletion and HDAC inhibition indicated that this combination may exert a strong antitumor effect. Cell viability assays revealed that although UHRF1 depletion induced only moderate or minimal growth suppression in DLD1 and RKO cells, adding TSA nearly completely suppressed the cell proliferation (Fig. 6a). EdU intake assays also showed that UHRF1 depletion alone induced moderate G1 phase arrest in DLD1 cells (Fig. 6b), but UHRF1 depletion plus TSA strongly induced arrest at G1 and G2/M phase in DLD1 and RKO cells (Fig. 6b; Additional file 1: Figure S9). We also carried out apoptosis assays in DLD1 cells and found that the incidence of apoptosis induced by TSA alone was similar to that induced by UHRF1 depletion plus TSA (Additional file 1: Figure S10).

**Fig. 7:**
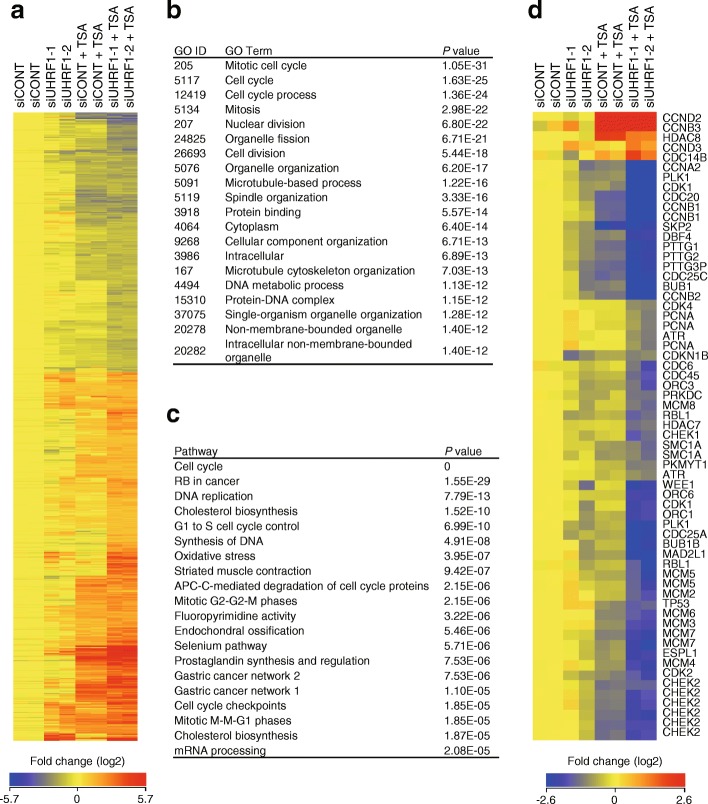
UHRF1 depletion and HDAC inhibition induce significant changes in the gene expression profiles of CRC cells. **a** Heat map showing the expression of genes altered by UHRF1 depletion and HDAC inhibition in DLD1 cells. Cells were transfected with the indicated siRNAs and incubated for 48 h, after which the cells were treated with or without TSA for 24 h, and gene expression microarray analysis was performed. **b** Gene ontology analysis of the selected genes shown in **a**. **c** Pathway analysis of the selected genes in **a**. **d** Heat map showing the expression of cell cycle-related genes

The above results suggest that depletion of UHRF1 plus HDAC inhibition suppresses CRC cell proliferation by inducing cell cycle arrest. To further clarify the underlying mechanism, we carried out the gene expression microarray analyses in DLD1 cells, with or without UHRF1 depletion and/or TSA treatment. The results showed that expression of 6190 probe sets (4498 unique genes) was significantly altered by UHRF1 depletion plus TSA as compared to control siRNA and mock treatment (> 2-fold and *P* < 0.05; Fig. 7a). By contrast, the effects of UHRF1 depletion alone on gene expression profiles were relatively limited (Fig. 7a). Gene ontology analysis showed that genes associated with “cell cycle” and “mitosis” were significantly enriched among genes affected by UHRF1 depletion plus HDAC inhibition (Fig. 7b). Similarly, pathway analysis suggested that genes involved in “cell cycle,” “RB in cancer,” and “DNA replication” were enriched among the affected genes (Fig. 7c; Additional file 1: Figure S11). Notably, we found that a number of cell cycle-related genes were significantly downregulated by UHRF1 depletion plus TSA (Fig. 7d). To validate the above results, we also performed a gene expression microarray analysis with RKO cells. Gene ontology and pathway analyses again revealed that genes associated with “cell cycle” were significantly enriched among those affected by UHRF1 depletion plus TSA (Additional file 1: Figure S12).

## Discussion

In the present study, we showed that UHRF1 depletion rapidly induces genome-wide DNA demethylation in cancer cells. Earlier studies showed that knockdown of DNMT1 or double knockdown of DNMT1 and DNMT3B induces DNA demethylation in various cancer cells [28–31]. Our results suggest that cancer cells also require UHRF1 to maintain DNA methylation. However, it is also noteworthy that the role of UHRF1 in DNA methylation may differ among tumor types. For instance, overexpression of UHRF1 causes genome-wide DNA hypomethylation in hepatocellular carcinoma and esophageal squamous cell carcinoma [12, 32], while UHRF1 has only minor effects on DNA methylation in retinoblastoma [33].

In our analysis, demethylation induced by UHRF1 depletion was observed across the entire genomic regions in CRC cells, including CpG islands, gene bodies, intergenic regions, and repetitive elements. This suggests UHRF1 is essential for maintaining DNA methylation in cancer cells and is generally more involved in the hypermethylation of tumor-related genes than the previously documented. UHRF1 depletion led to demethylation in the CpG islands of well-known tumor-related genes in both CIMP-positive and CIMP-negative CRC cell lines, as well as in a breast cancer cell line. However, it was unexpected that hypermethylated genes would continue to be repressed after UHRF1 depletion, as it is well documented that UHRF1 acts as a hub to recruit multiple proteins, including DNMT1, HDAC1, G9a, and EZH2, to repress cancer-associated genes in cancer cells [15, 21, 22, 34].

Notably, we found that UHRF1 depletion plus HDAC inhibition restored the expression of a number of tumor suppressor genes in CRC cells. *SFRP* family genes and *DKK1* encode secreted Wnt inhibitor proteins and are frequently silenced via CpG island hypermethylated in CRC [35–37]. The *GATA4* and *GATA5* transcription factor genes are potential tumor suppressor genes and are frequently hypermethylated in CRC [38, 39]. Hypermethylation of *NEURL* was discovered in a screen to identify epigenetically silenced genes in CRC cells [25]. Much experimental evidence suggests that re-expression of these genes suppresses CRC cell proliferation [25, 35–37, 39]. *UCHL1*, also known as *PGP9.5*, is prevalently methylated in various cancers, including CRC [40], and its tumor suppressor function has been experimentally demonstrated [41]. These results suggest that the antitumor effect of UHRF1 depletion plus HDAC inhibition is due, at least in part, to the restored expression of multiple tumor suppressor genes.

The effect of UHRF1 depletion plus HDAC inhibition to reverse gene silencing may suggest that UHRF1 depletion alone is not sufficient to induce histone acetylation at the hypermethylated genes. Those findings are reminiscent of an earlier report showing that low-dose 5-aza-dC and TSA synergistically restored the expression of hypermethylated genes in cancer cells [42]. Although URHF1 mediates cross-talk between DNA methylation and histone acetylation through interaction with DNMT1 and HDAC1 [4], HDACs may nonetheless be recruited to the silenced genes in the UHRF1-depleted cancer cells. Moreover, because the inhibition of UHRF1 leads to DNA demethylation by suppressing the recruitment of DNMT1 to newly synthesized hemi-methylated DNA, substantial numbers of cancer cells retain hemi-methylated DNA after UHRF1 depletion. Our bisulfite sequencing analysis revealed that UHRF1-depleted cells exhibit a mixture of fully methylated and significantly demethylated DNA. Even in the demethylated alleles, small numbers of CpG sites remained methylated. Thus, the residual DNA methylation may be sufficient to be recognized by methyl-DNA-binding proteins that recruit HDACs.

One recent study demonstrated an interesting antagonistic role of UHRF1 in mice. Sharif et al. showed that conditional knockout of either *Dnmt1* or *Uhrf1* leads to significant genome-wide demethylation in mouse embryos and embryonic stem cells, but only *Dnmt1*-ablated cells show derepression of endogenous retroviruses [43]. They demonstrated that, in the absence of DNMT1, UHRF1 is paradoxically required for the activation of endogenous retroviruses and acts through binding to hemi-methylated DNA and disrupting histone H3 lysine 9 (H3K9) tri-methyltransferase SETDB1-mediated silencing. Their results suggested that both DNMT1 and UHRF1 are essential for maintaining DNA methylation, while UHRF1 plays an opposite role in different repressive pathways. It is also possible that UHRF1 has similarly complex actions in cancer cells, and further study is warranted.

Earlier studies suggest UHRF1 is required for cell cycle progression. By downregulating pRB at the protein and gene transcription levels, UHRF1 promotes G1/S transition in human lung fibroblasts and Jurkat cells [9]. Upon DNA damage, expression of UHRF1 is suppressed by the p53/p21 signal, and knockdown of UHRF1 induces G1 arrest after DNA damage in HeLa cells [44]. By contrast, depletion of UHRF1 induces G2/M phase arrest in a CRC cell line HCT116 [18]. We found that UHRF1 depletion induces moderate G1 arrest in DLD1 cells, while UHRF1 depletion plus HDAC inhibition strongly induces G1 and G2/M arrest. Although it is well known that TSA blocks the cell cycle by inducing p21 [45], a strong induction of p21 was not observed in CRC cells with UHRF1 depletion plus TSA (data not shown). Instead, we found that UHRF1 depletion plus TSA induced marked changes in the gene expression profiles in CRC cells and that cell cycle-related genes were strikingly enriched among the downregulated genes. Many of these genes were moderately downregulated by either one of the treatments, but the combination treatment strongly blocked the cell cycle.

Our results may also have clinical implications, as a number of studies have shown UHRF1 to be a potential therapeutic target in human malignancies [46, 47]. In normal colonic mucosa, UHRF1 is expressed in the proliferative compartment of colonic crypts and is co-expressed with a proliferative marker, Ki-67. UHRF1 expression is elevated in approximately 60% of CRC tissues and is associated with decreased expression of its target gene, PPARG [20, 48]. Earlier studies also showed the growth suppressive effects of UHRF1 knockdown in CRC cells [16, 20, 48, 49]. Several natural compounds are known to exert antitumor effects by downregulating UHRF1 [47, 50]. For instance, hinokitiol (4-isopropyltropolone), a component of essential oils extracted from the Japanese cypress (*Chamaecyparis obtuse*), induces DNA demethylation via DNMT1 and UHRF1 inhibition in CRC cells [50]. In addition, a green tea polyphenol, epigallocatechin 3-gallate, contributes to the degradation of DNMT3A and HDAC3 in CRC cells, at least in part, by inhibiting their interaction with UHRF1 [51]. Other studies also showed that inhibiting UHRF1 enhances the chemosensitivity in breast cancer and radiosensitivity in esophageal squamous cell carcinoma [19, 52]. In that context, our present findings suggest UHRF1 may be an effective therapeutic target for sensitizing cancer cells to antitumor agents. However, it is noteworthy that our experimental conditions, which included high doses of TSA and 5-aza-dC, are significantly cytotoxic. In addition, disruption of the DNMT1/PCNA/UHRF1 complex in normal cells reportedly induces global DNA hypomethylation and tumorigenesis [53]. Thus, further study is necessary to clarify the clinical usefulness and safety of therapy targeting UHRF1.

## Conclusions

In summary, we observed that UHRF1 depletion plus HDAC inhibition effectively restores the expression of genes epigenetically silenced in CRC cells. We also demonstrated that HDAC inhibition strongly suppresses proliferation of UHRF1-depleted CRC cells. These findings suggest that a closed chromatin state persists after demethylation induced by UHRF1 depletion in cancer cells and that dual targeting of UHRF1 and histone modifiers may restore the expression of epigenetically silenced genes.

## Methods

### Cell lines

CRC cell lines (CaCO2, Colo320, DLD1, HCT116, HT29, LoVo, RKO, SW48, SW480, SW620, T84, and WiDr), a breast cancer cell line (MCF7), and HEK293 cells were described previously [54–56]. DLD1, RKO, MCF7, and HEK293 cells were maintained in DMEM supplemented with 10% fetal bovine serum. SW480 cells were maintained in McCoy’s 5A medium supplemented with 10% fetal bovine serum. Cells were tested for mycoplasma contamination. Genomic DNA was extracted using the standard phenol-chloroform method. Total RNA was extracted using TRI Reagent (COSMO BIO, Tokyo, Japan). Total RNA samples from normal colonic tissues were purchased from BioChain (Newark, CA, USA) and Thermo Fisher Scientific (Waltham, MA, USA).

### Transfection of siRNA and drug treatment

For RNA interference-induced knockdown of UHRF1, cells (5 × 10^5^ cells in 6-well plate) were transfected with 25 pmol of Silencer Select Pre-designed siRNA (siUHRF1-1, s26553; siUHRF1-2, s26554, Thermo Fisher Scientific) or a Silencer Select Negative Control No. 1 siRNA (Thermo Fisher Scientific) using Lipofectamine RNAiMAX (Thermo Fisher Scientific) according to the manufacturer’s instructions. Cells were harvested 72 h or 96 h after transfection. For combined UHRF1 knockdown and HDAC inhibition, cells transfected with siRNA were incubated for 48 h or 72 h, after which the transfectants were treated with 300 nM trichostatin A (TSA) or mock (ethanol) for an additional 24 h. As a control for gene reactivation through DNA demethylation, cells were treated with 1 μM 5-aza-2′-deoxycytidine (5-aza-dC) for 72 h, replacing the drug and medium every 24 h.

### Reverse transcription PCR

Reverse transcription PCR (RT-PCR) was carried out as described previously [54]. Glyceraldehyde-3-phosphate dehydrogenase (*GAPDH*) and β-actin (*ACTB*) were used as endogenous controls. qRT-PCR of tumor-related genes was carried out using TaqMan Gene Expression Assays for *UHRF1*, Hs01086727_m1; *GAPDH*, Hs02758991_g1; *ACTB*, Hs01060665_g1; *DKK1*, Hs00183740_m1; *GATA4*, Hs00171403_m1; *GATA5*, Hs00388359_m1; *MLH1*, Hs00179866_m1; *NEURL*, Hs00184868_m1; *SFRP1*, Hs00610060_m1; *SFRP2*, Hs00293258_m1; *SFRP5*, Hs00169366_m1; *TIMP3*, Hs00165949_m1; and *UCHL1*, Hs00985157_m1 (Thermo Fisher Scientific) with a 7500 Fast Real-Time PCR System (Thermo Fisher Scientific). qRT-PCR analysis of CIMP marker genes was carried out using PowerUp SYBR Green PCR Master Mix (Thermo Fisher Scientific). Primer sequences and PCR product sizes are listed in Additional file 2: Table S1.

### Western blot analysis

Western blot analysis was performed as described previously [57]. Mouse anti-UHRF1 (1:500 dilution, catalog no 612264, BD Transduction Laboratories, Franklin Lakes, NJ, USA) and anti-β-actin (1:10000 dilution, clone AC-15, Sigma-Aldrich, St. Louis, MO, USA) monoclonal antibodies were used.

### Dot blot analysis

Dot blot analysis was performed as described previously [56]. A mouse anti-5-methylcytosine (5-mC) monoclonal antibody (1:1000 dilution, catalog no 39649, Active Motif, Carlsbad, CA, USA) was used.

### DNA methylation analysis

Genomic DNA was modified with sodium bisulfite using an EpiTect Bisulfite Kit (Qiagen, Hilden, Germany), after which bisulfite pyrosequencing and bisulfite sequencing were carried out as described previously [54]. Primer sequences and PCR product sizes are listed in Additional file 2: Table S1. Primer sequences for LINE1, Alu Yb8, and NBL2 were described previously [58].

### Infinium assay

Genome-wide DNA methylation was analyzed using an Infinium HumanMethylation450 BeadChip according to the manufacturer’s instructions (Illumina, San Diego, CA, USA), as described previously [56]. The Gene Expression Omnibus accession number for the Infinium assay data is GSE106439.

### Chromatin immunoprecipitation PCR

Chromatin immunoprecipitation (ChIP) was carried out using an anti-acetyl-histone H3 lysine 9 antibody (#07-352, Millipore, Billerica, MA, USA) as described previously [59]. Input DNA and the immunoprecipitate were subjected to qRT-PCR analysis using PowerUp SYBR Green PCR Master Mix (Thermo Fisher Scientific). Primer sequences and PCR product sizes are listed in Additional file 2: Table S1.

### Cell viability assays

Cells (5 × 10^3^ cells/well in a 96-well plate) were transfected with 1 pmol of siRNA using Lipofectamine RNAiMAX and incubated for 48 h as described above. The transfectants were then treated with 300 nM TSA or mock (ethanol) for an additional 24 h. Cell viability assays were carried out using a Cell Counting Kit-8 (Dojindo, Kumamoto, Japan) according to the manufacturer’s instructions.

### Cell proliferation assays

EdU cell proliferation assays were performed using a Click-iT Plus EdU Alexa Fluor 647 Flow Cytometry Assay Kit (Thermo Fisher Scientific). Briefly, 1 × 10^6^ cells were incubated with 10 μM EdU for 2 h, after which the cells were fixed with Click-iT fixative. After washing, the cells were treated with Click-iT plus reaction cocktail, and Alexa Fluor 647-labeled cells were analyzed using a BD FACSCanto II (BD Biosciences, Franklin Lakes, NJ, USA) with BD FACSDiva software (BD Biosciences). Data were analyzed using FlowJo version 10 (Tree Star Inc., Ashland, OR, USA).

### Apoptosis assay

Apoptosis was analyzed using an ApoScreen Annexin V Apoptosis Kit (SouthernBiotech, Birmingham, AL, USA). Briefly, 1 × 10^6^ cells were washed twice with cold PBS and stained with annexin V-FITC and propidium iodide (PI). The stained cells were analyzed using a BD FACSCanto II (BD Biosciences) running BD FACSDiva software (BD Biosciences). Data were analyzed using FlowJo version 10 (Tree Star Inc.).

### Gene expression microarray

Gene expression microarray analysis was carried out using SurePrint G3 Human GE microarray v2 according to the manufacturer’s instructions (Agilent Technologies, Santa Clara, CA, USA), as described previously [56]. The microarray data were analyzed using GeneSpring GX version 13 (Agilent Technologies). The Gene Expression Omnibus accession number for the microarray data is GSE106439.

### Statistical analysis

Comparisons of continuous variables were made using *t* tests or one-way ANOVA with post hoc multiple comparisons (Tukey HSD test). Values of *P* < 0.05 (two-sided) were considered significant. Data were analyzed using GraphPad Prism 5 (GraphPad Software, La Jolla, CA, USA).

## Additional files

Additional file 1: Supplementary figures. (DOC 3751 kb)

Additional file 2: Supplementary **Table S1.** (XLS 35 kb)

## Abbreviations

5-aza-dC: 5-Aza-2′-deoxycytidine
5-mC: 5-Methylcytosine
CIMP: CpG island methylator phenotype
CRC: Colorectal cancer
DKK1: Dickkopf 1
DNMT: DNA methyltransferase
EdU: 5-Ethynyl-2′-deoxyuridine
GAPDH: Glyceraldehyde-3-phosphate dehydrogenase
MLH1: MutL homolog 1
RB: Retinoblastoma
SD: Standard deviation
HDAC: Histone deacetylase
SFRP: Secreted frizzled-related protein
SRA: SET and RING finger-associated
TCGA: The Cancer Genome Atlas
TSA: Trichostatin A
UHRF1: Ubiquitin-like protein containing PHD and RING finger domains 1
UCHL1: Ubiquitin carboxyl-terminal esterase l
