# Supplementary material for: UHRF1 depletion and HDAC inhibition reactivate epigenetically silenced genes in colorectal cancer cells
Source: Clin Epigenetics. 2019 May 7;11:70. doi: 10.1186/s13148-019-0668-3 (PMC6505222; doi:10.1186/s13148-019-0668-3)
Supplement: Supplementary file 1 — Supplementary figures. (DOC 3751 kb) [file 13148_2019_668_MOESM1_ESM.doc]

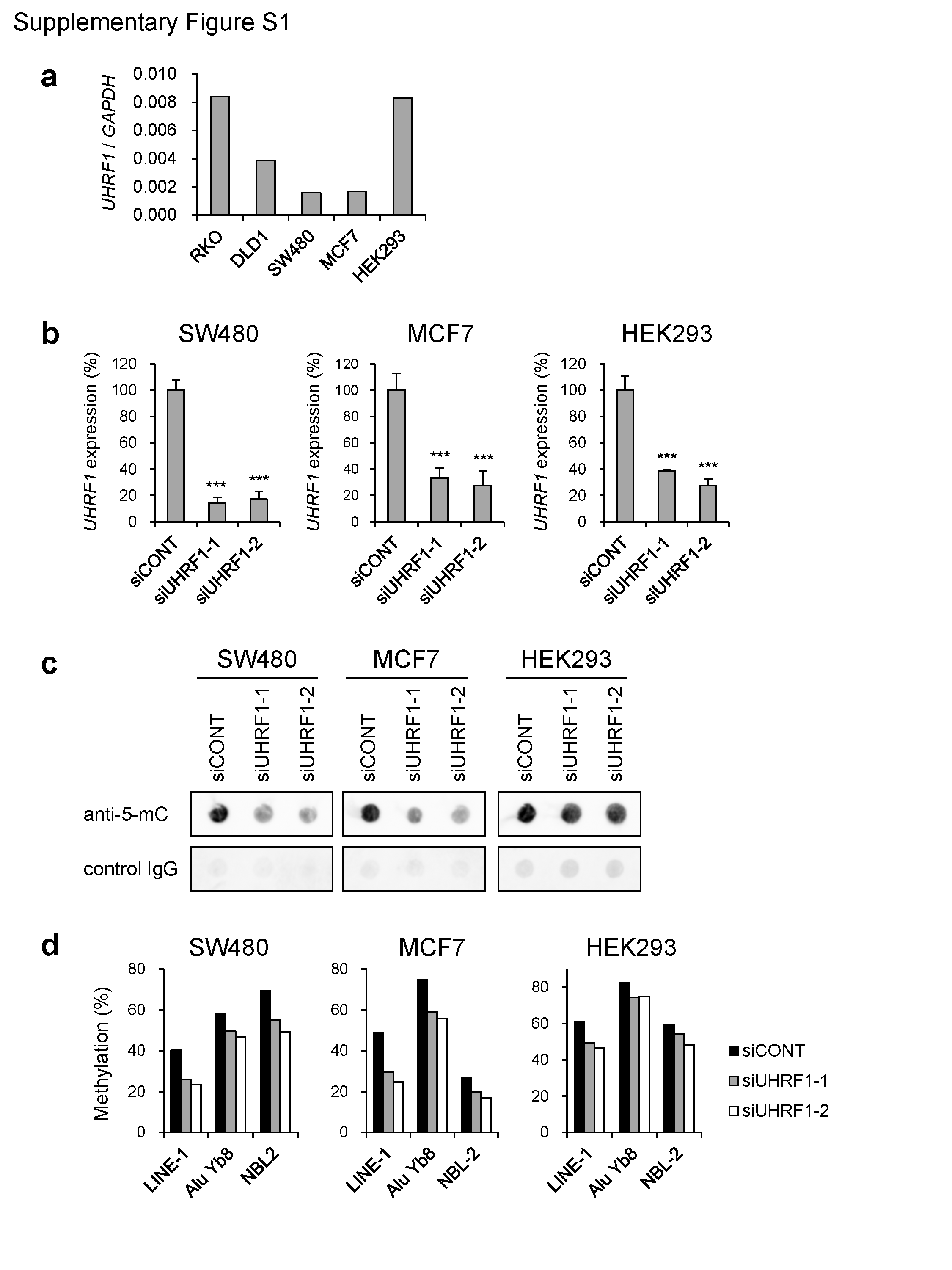


**Figure S1**. UHRF1 depletion induces global DNA demethylation in cancer cells. (a) qRT-PCR analysis of *UHRF1* in cancer cell lines and HEK293 cells. (b) qRT-PCR analysis showing *UHRF1* knockdown in SW480, MCF7 and HEK293 cells. Cells were transfected with a control siRNA (siCONT) or siRNAs targeting UHRF1 and were harvested 96 h after transfection. Shown are means of 3 replications; error bars represent SDs. ****P* < 0.001. (c) Dot blot analysis of 5-methylcytosine (5-mC) in CRC cells transfected with the indicated siRNAs. Cells were transfected with the indicated siRNAs, and genomic DNA was extracted 96 h after transfection. Results using control IgG are shown as loading controls. (d) Bisulfite pyrosequencing of repetitive elements in CRC cells transfected with the indicated siRNAs.


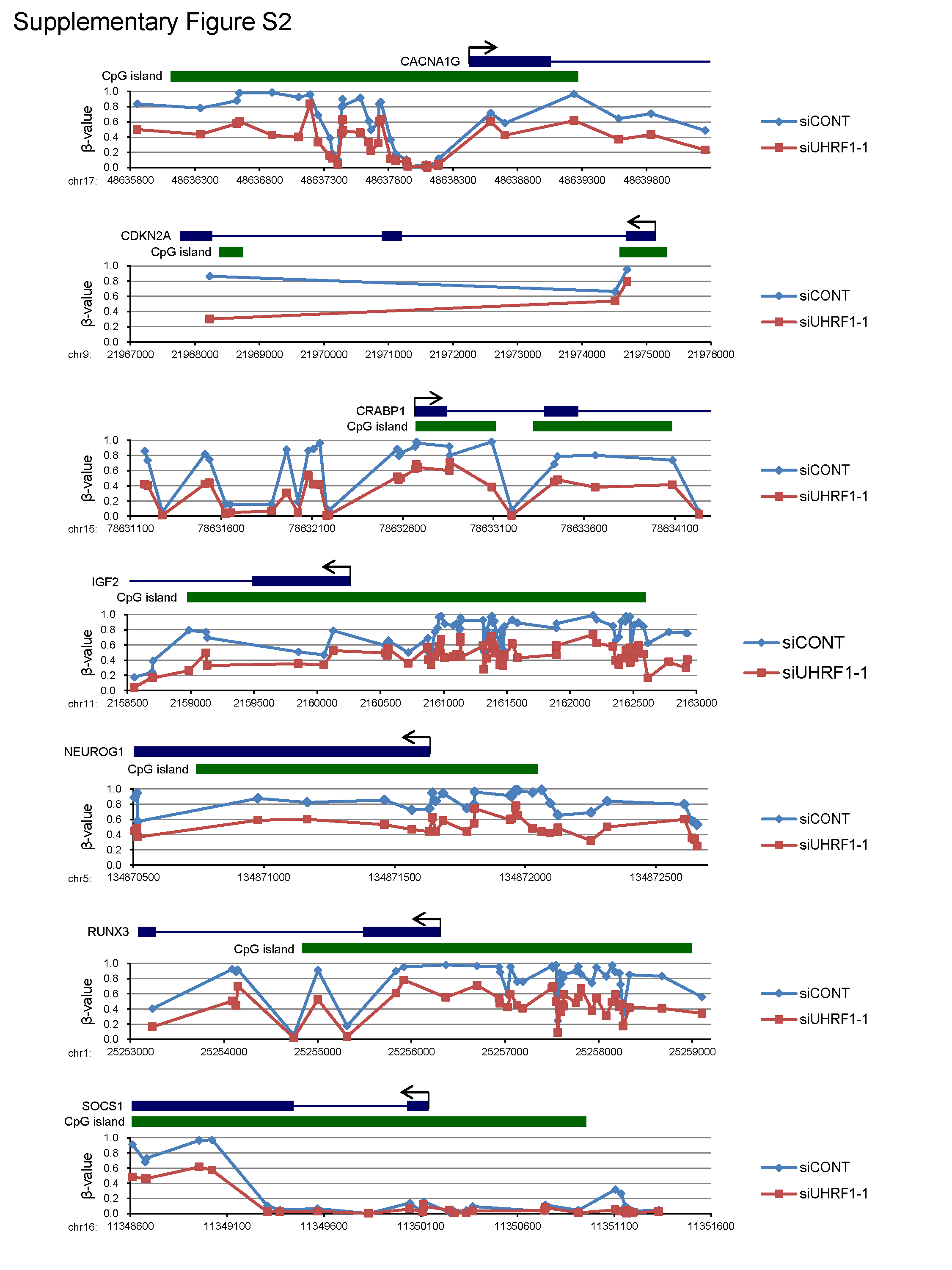


**Figure S2**. Results of BeadChip assays showing demethylation of CIMP marker genes in DLD1 cells after UHRF1 knockdown. Shown are β-values of BeadChip probes located in the promoter regions of the genes. Locations of the CpG islands and the first exons are indicated at the top.


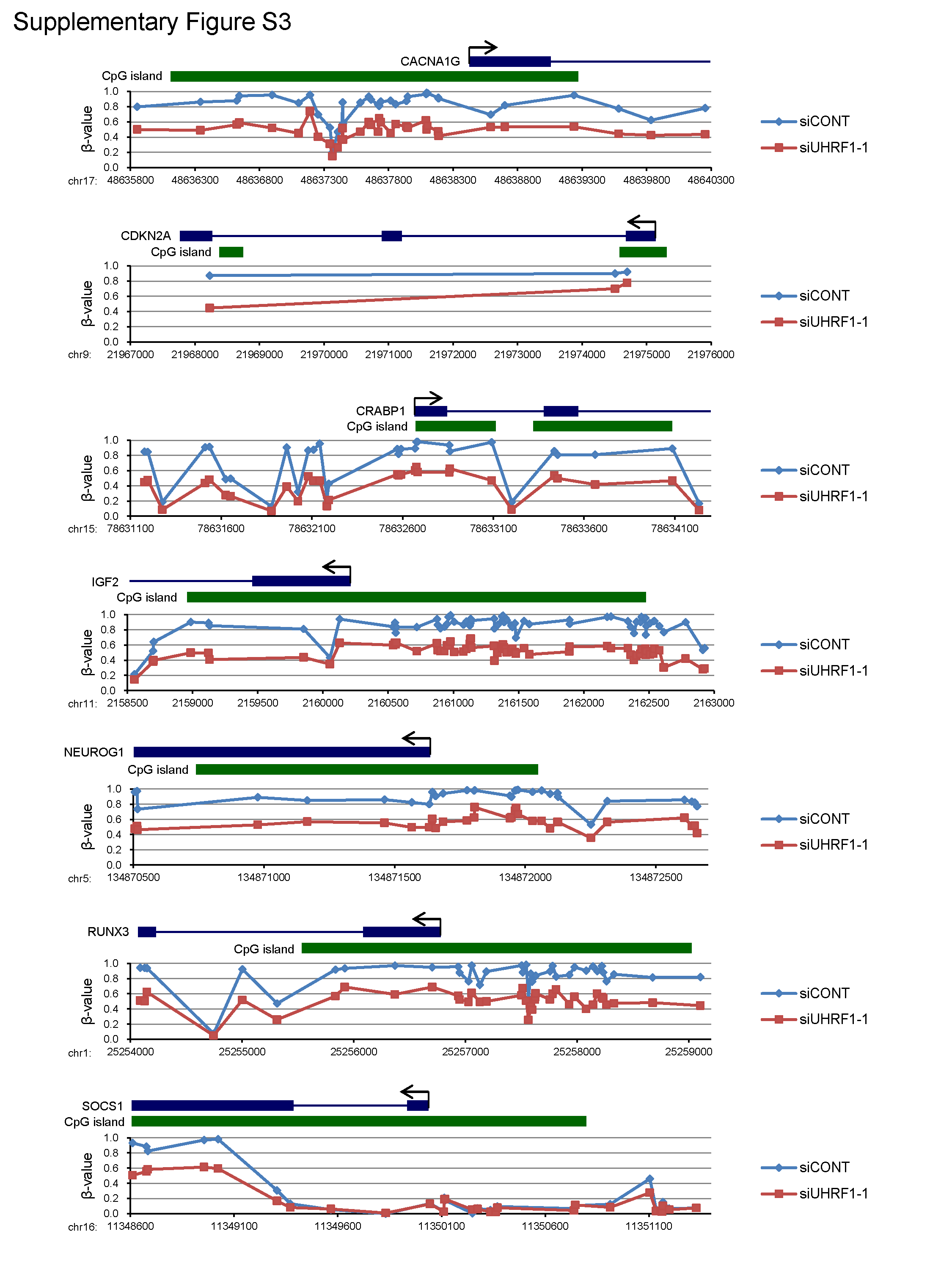


**Figure S3**. Results of BeadChip assays showing demethylation of CIMP marker genes in RKO cells after UHRF1 knockdown. Shown are β-values of BeadChip probes located in the promoter regions of the genes. Locations of the CpG islands and the first exons are indicated at the top.


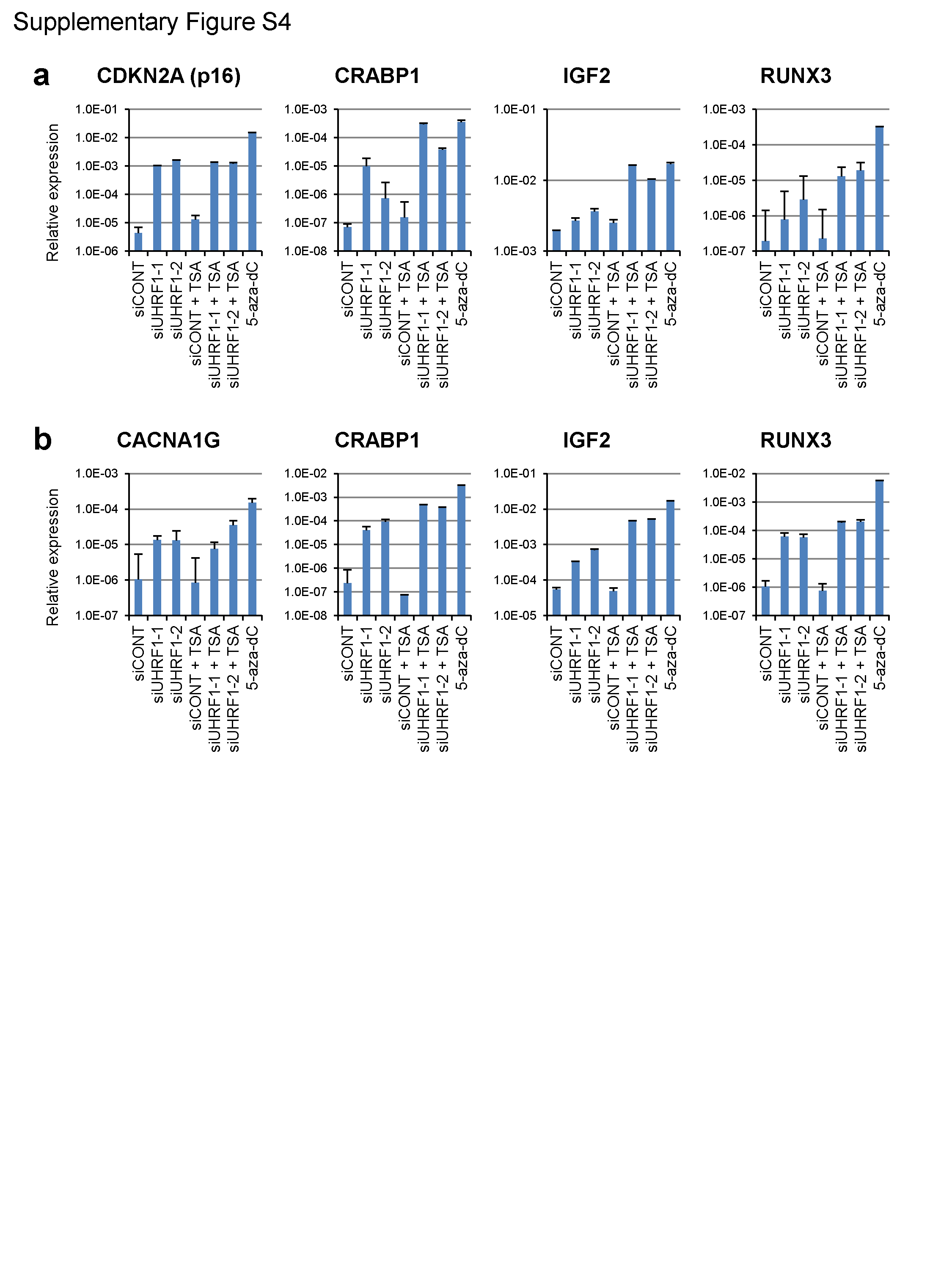


**Figure S4**. qRT-PCR analysis of CIMP marker genes in DLD1 (a) and RKO (b) cells. Cells were transfected with the indicated siRNAs and incubated for 48 h (DLD1) or 72 h (RKO), after which they were incubated with or without TSA for 24 h. Cells treated with 5-aza-dC are shown as positive controls for gene reactivation. Results are normalized to *ACTB* expression. Shown are means of 3 replications; error bars represent SDs.


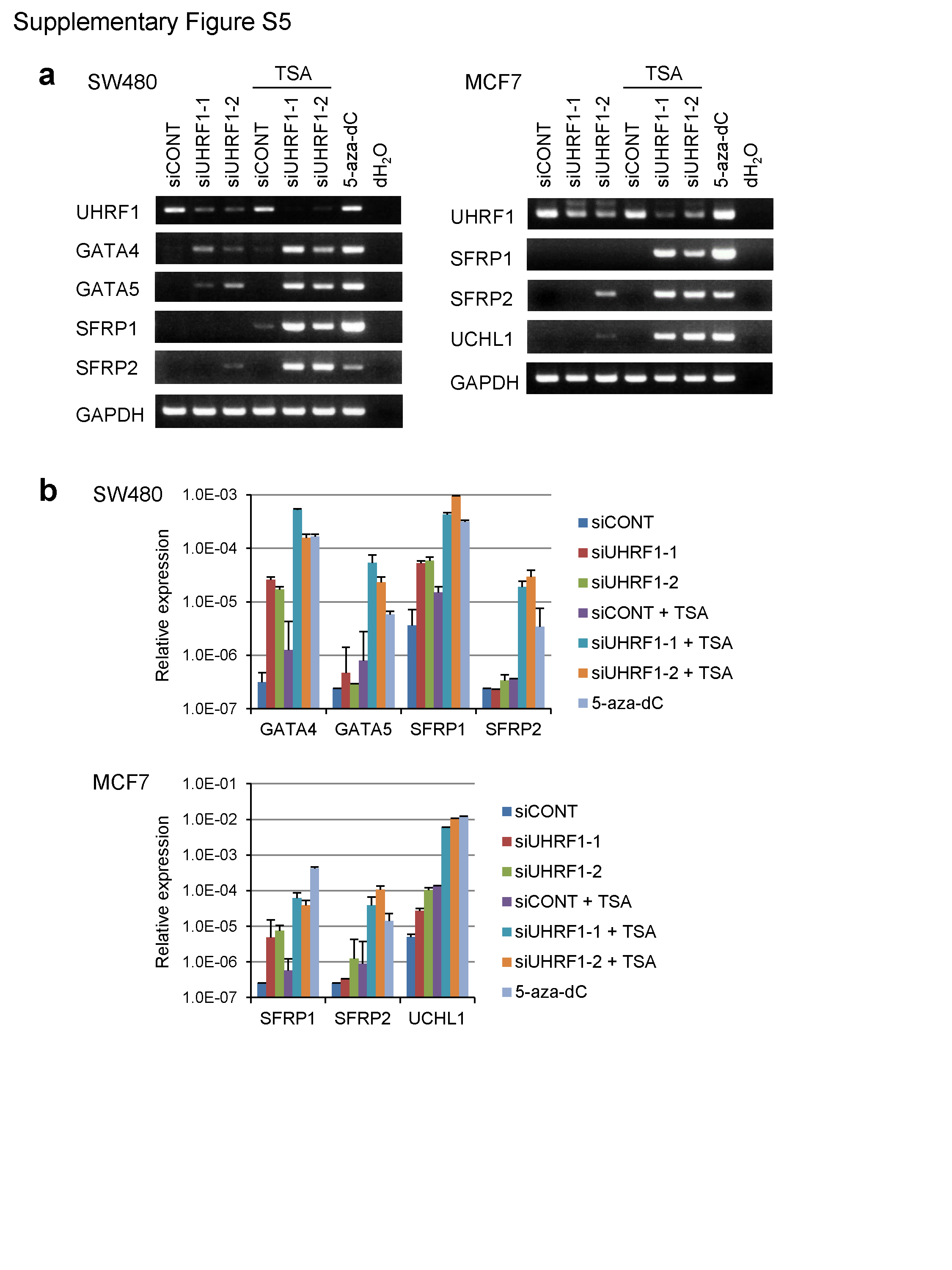


**Figure S5**. UHRF1 depletion plus HDAC inhibition restore expression of epigenetically silenced genes in cancer cells. (a) RT-PCR analysis of *UHRF1* and epigenetically silenced genes in SW480 and MCF7 cells. Cells were transfected with the indicated siRNAs and incubated for 72 h, after which they were treated with or without TSA for 24 h. Cells treated with 5-aza-dC are shown as positive controls for gene reactivation. (b) qRT-PCR analysis of epigenetically silenced genes in the same samples used in (a). Results are normalized to *GAPDH* expression.


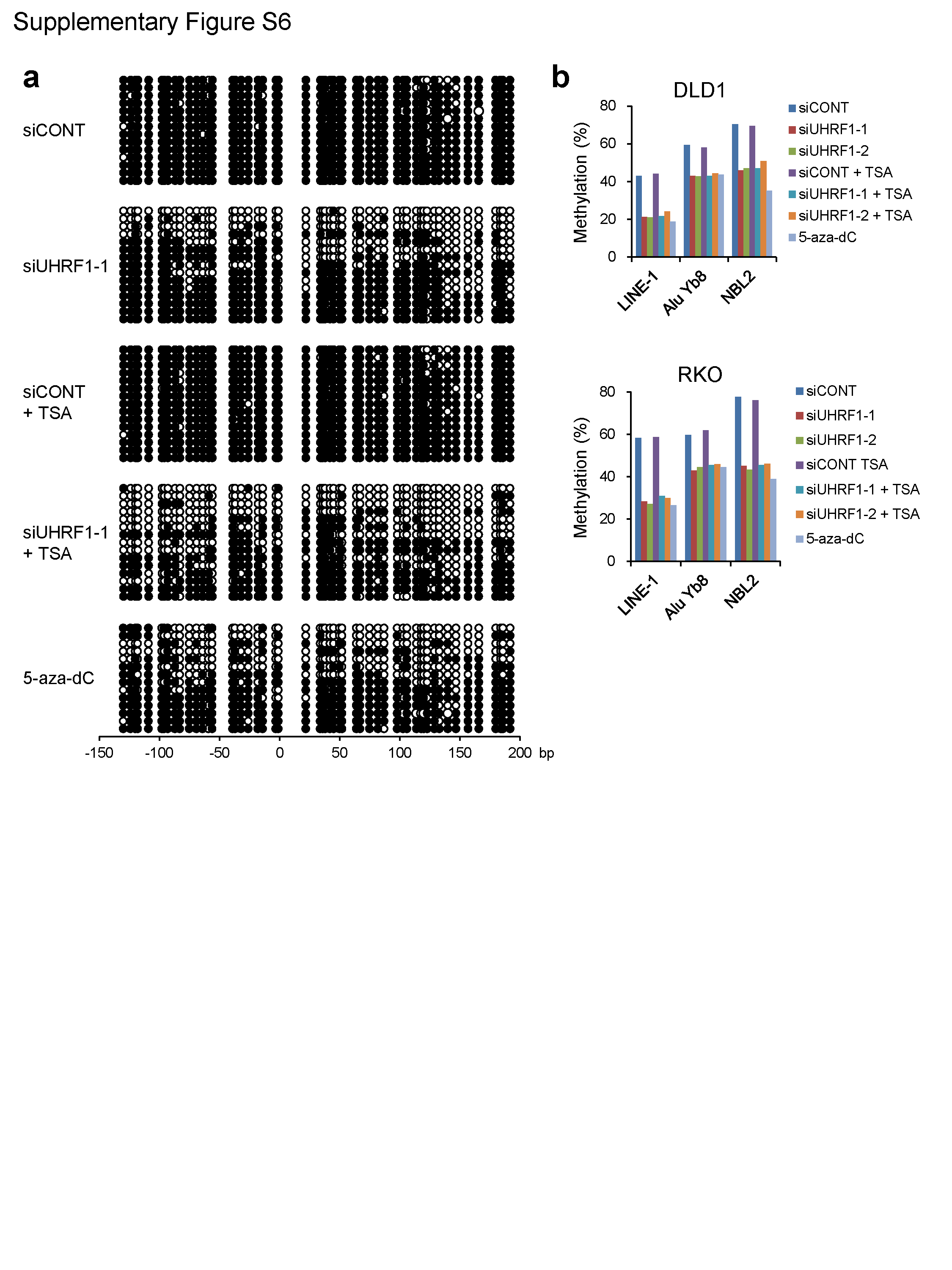


**Figure S6**. Additional HDAC inhibition in UHRF1-depleted CRC cells does not induce further DNA demethylation. (a) Bisulfite sequencing analysis of the *SFRP1* CpG island in RKO cells with the indicated siRNAs and treatment. Open and filled circles depicted unmethylated and methylated CpG sites, respectively. Locations relative to the transcription start site are shown below. (b) Bisulfite pyrosequencing analysis of repetitive elements in DLD1 and RKO cells with the indicated siRNAs and treatment.


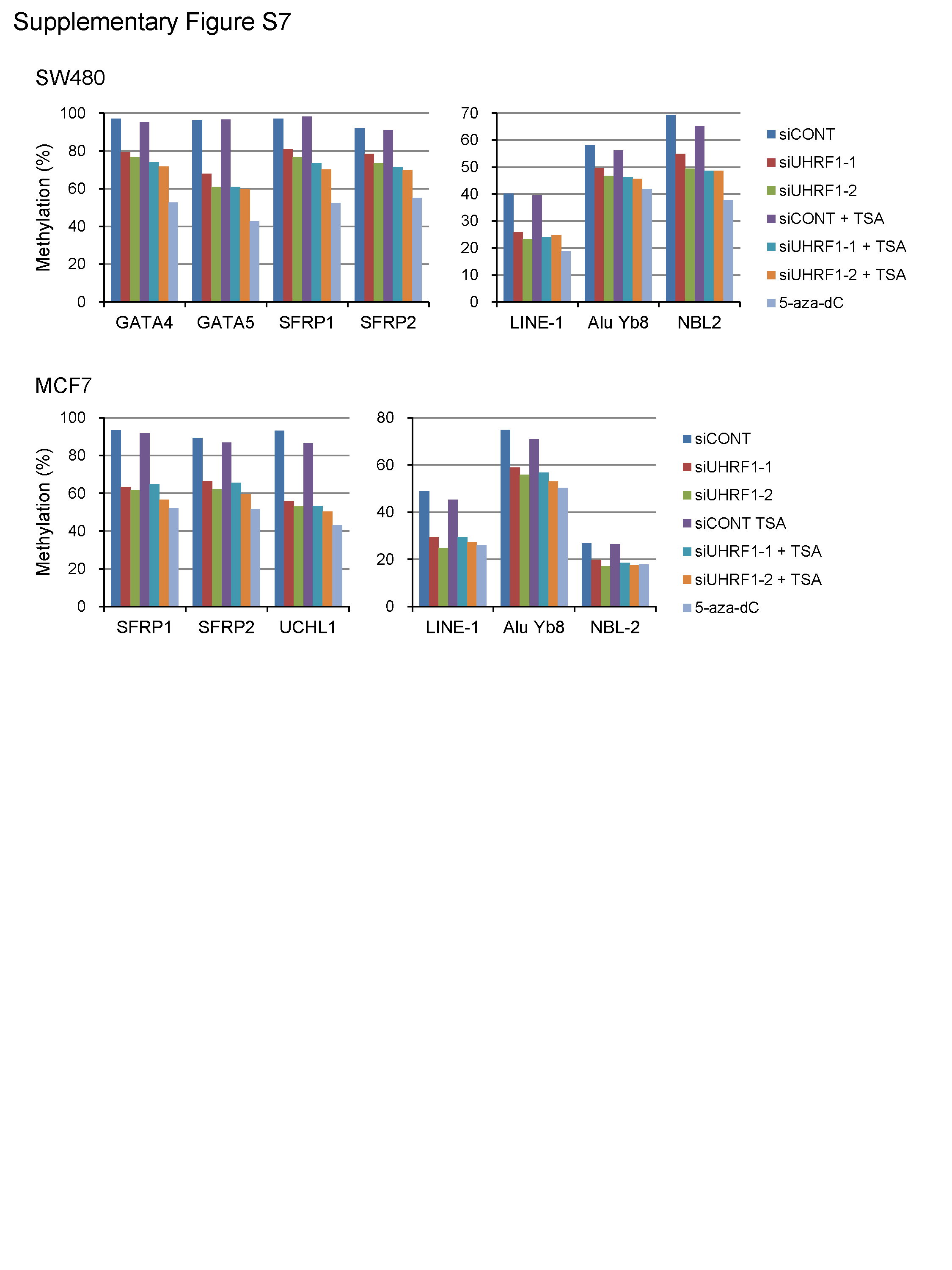


**Figure S7**. Bisulfite pyrosequencing analysis of epigenetically silenced genes (left) and repetitive elements (right) in SW480 and MCF7 cells. Cells were transfected with the indicated siRNAs and incubated for 72 h, after which they were treated with or without TSA for 24 h. Cells treated with 5-aza-dC are shown as positive controls for demethylation.


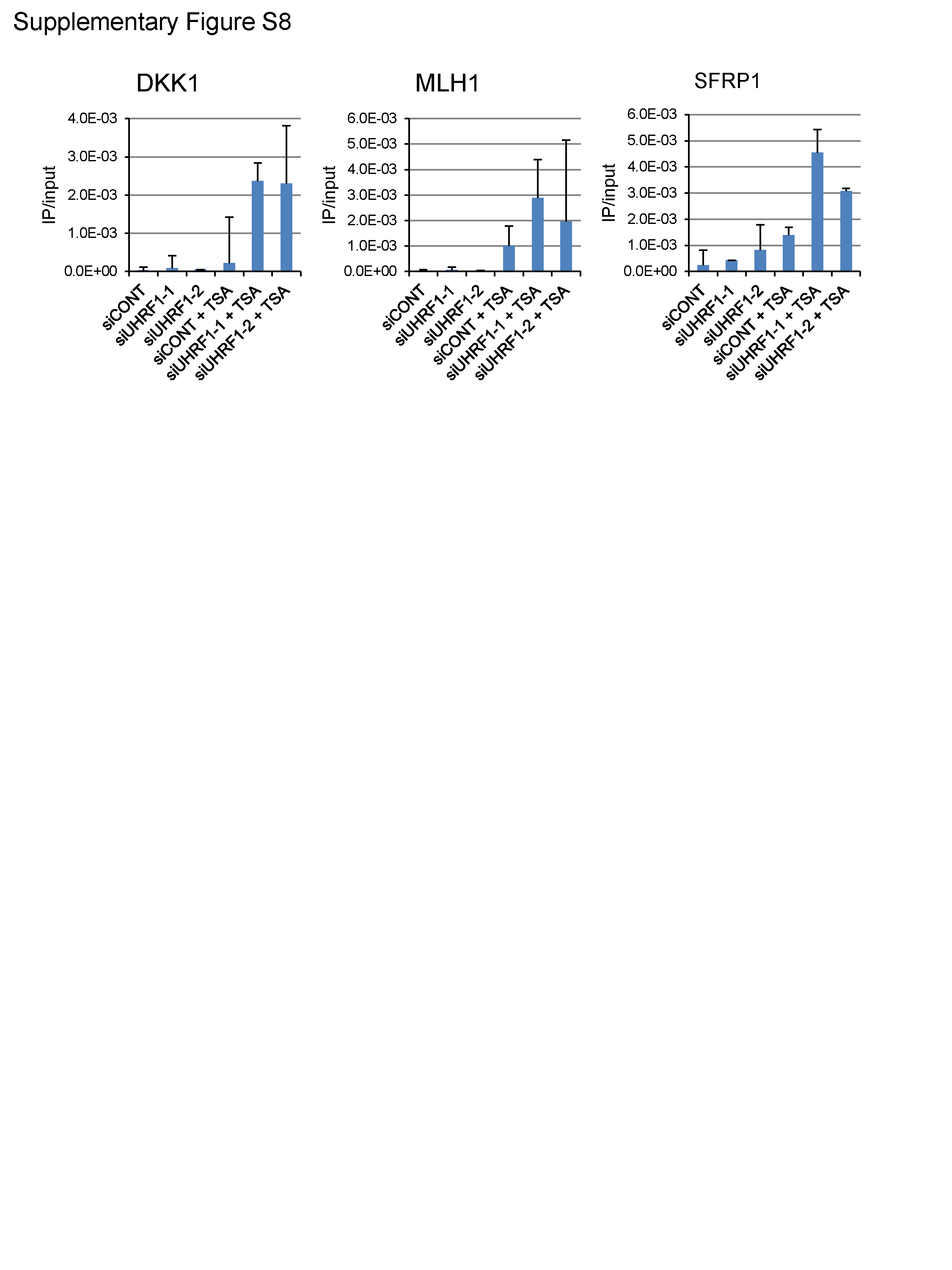


**Figure S8**. Chromatin immunoprecipitation (ChIP)-PCR analysis showing levels of H3K9ac at CpG islands of the indicated genes in RKO cells. Shown are means of 3 replications; error bars represent SDs.


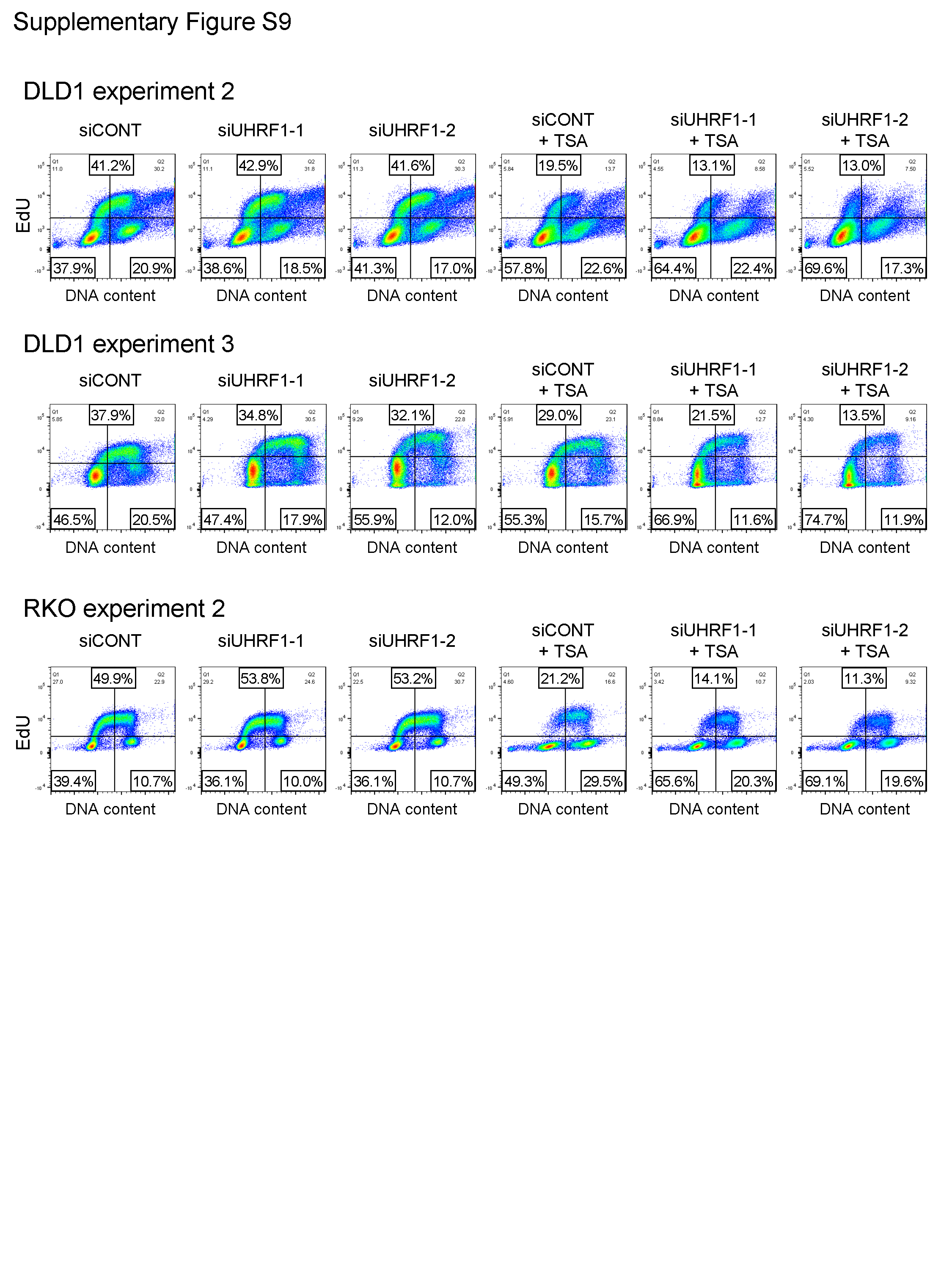


**Figure S9**. EdU cell proliferation assays in DLD1 and RKO cells treated with the indicated siRNAs alone or plus TSA. Results shown in Figure 6b were confirmed in independent experiments. Cells were transfected with the indicated siRNAs and incubated for 48 h, after which they were incubated with or without TSA for 24 h.


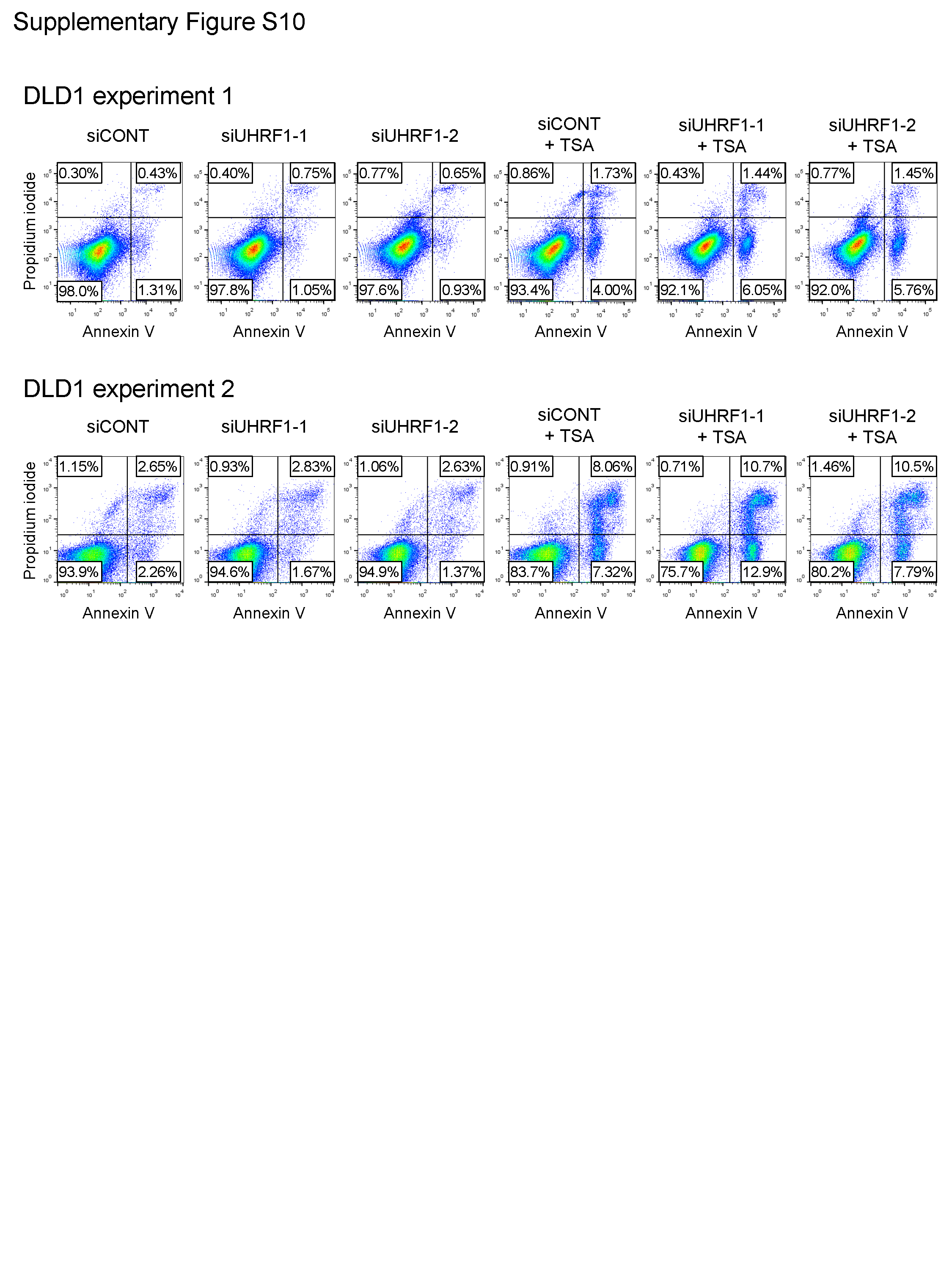


**Figure S10**. Results of apoptosis analysis in DLD1 cells. Cells were transfected with the indicated siRNAs and incubated for 48 h, after which they were incubated with or without TSA for 24 h. Results were confirmed in two independent experiments.


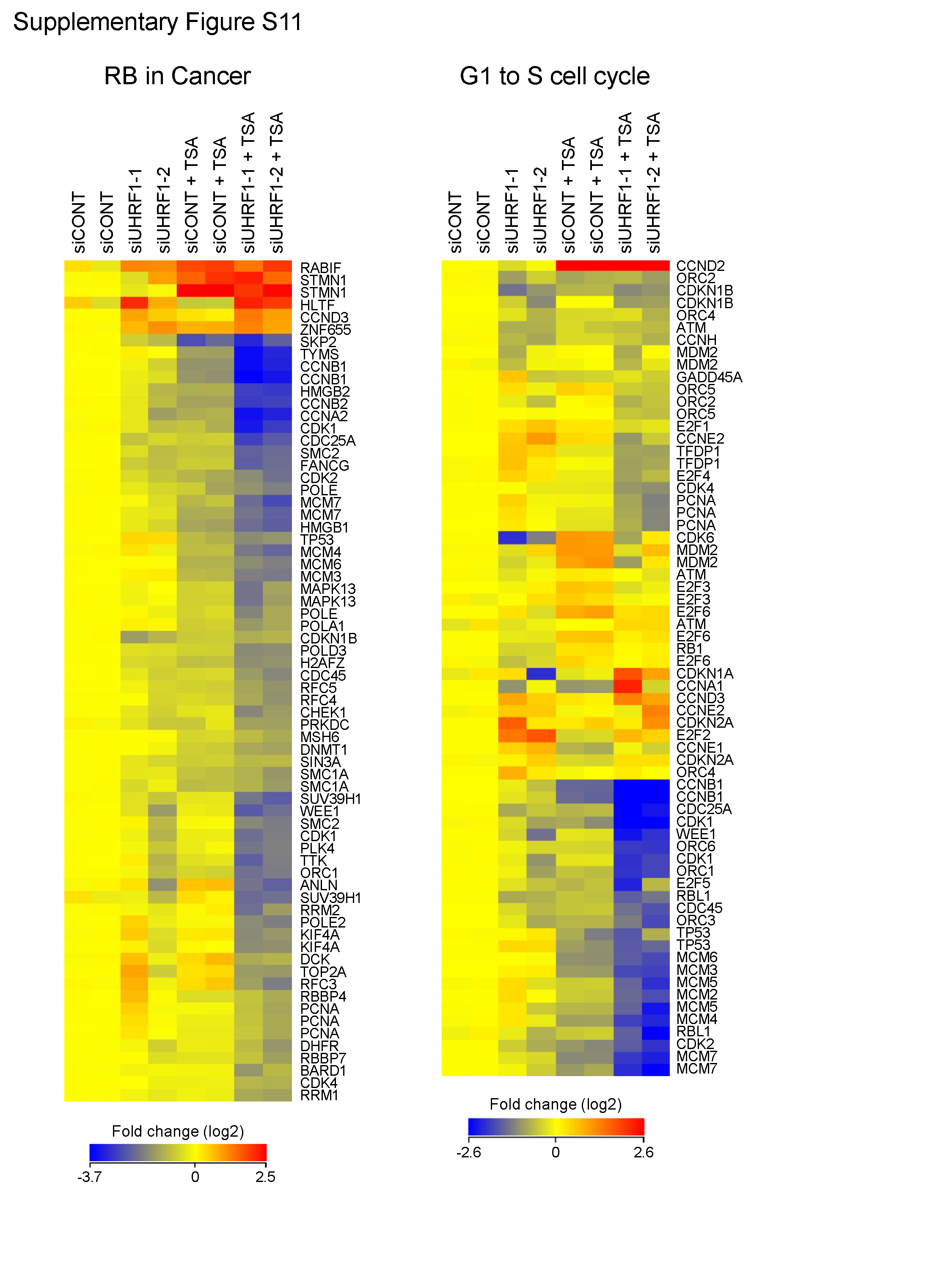


**Figure S11**. Cell cycle-related genes are affected by UHRF1 depletion plus HDAC inhibition in DLD1 cells. Heat maps showing genes associated with “RB in Cancer” and “G1 to S cell cycle” in DLD1 cells. Cells were transfected with the indicated siRNAs and incubated for 48 h, after which they were treated with or without TSA for 24 h, and gene expression microarray analysis was performed.


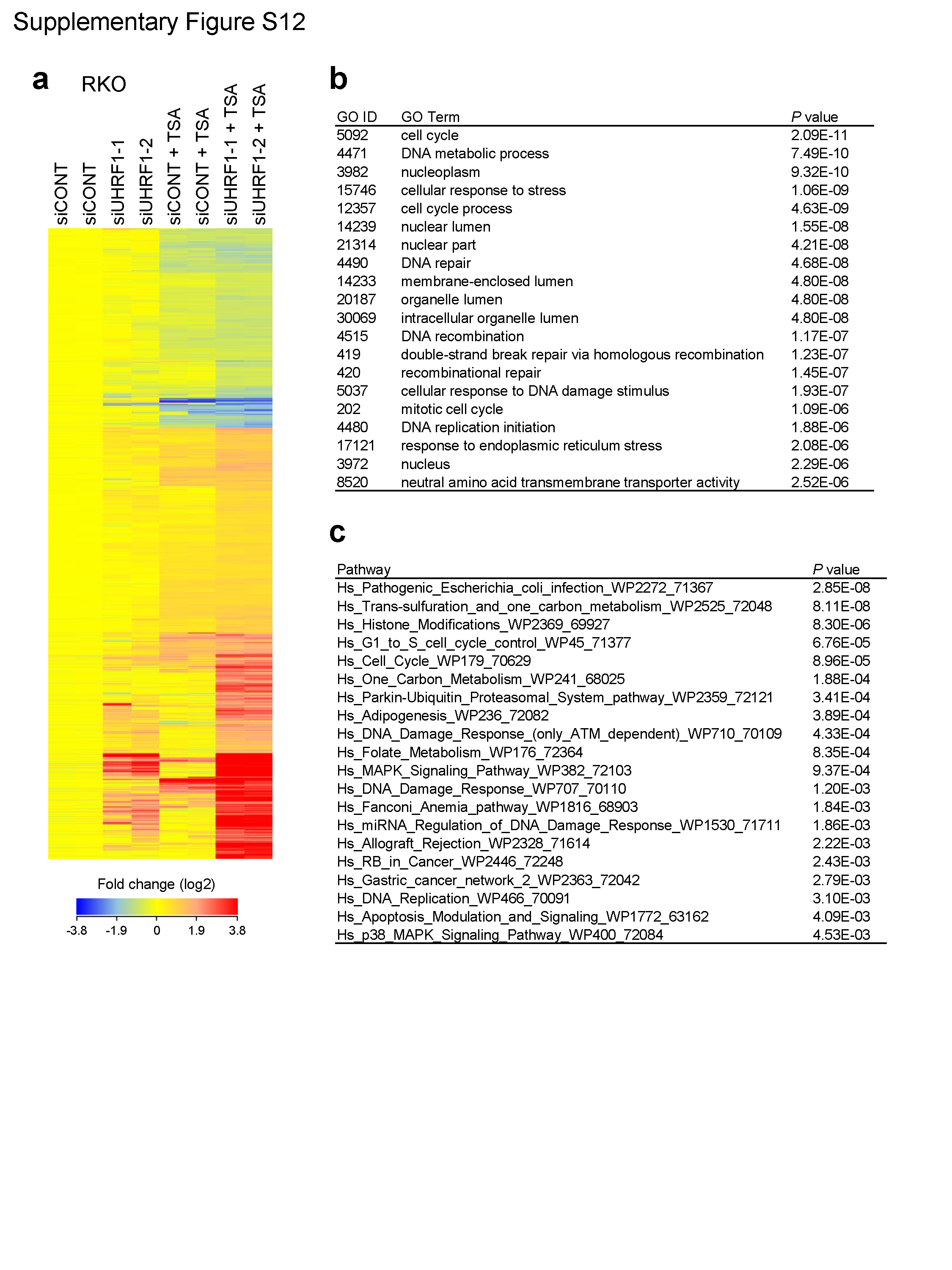


**Figure S12**. UHRF1 depletion and HDAC inhibition induce significant changes in the gene expression profiles in RKO cells. (a) Heat map showing expression of genes altered by UHRF1 depletion and HDAC inhibition in RKO cells. Cells were transfected with the indicated siRNAs and incubated for 72 h, after which the cells were incubated with or without TSA for 24 h and gene expression microarray analysis was performed. Genes whose expression was altered by UHRF1 knockdown and TSA were selected (*P* < 0.05, 2085 probe sets, 1793 unique genes), and hierarchical clustering was performed. (b) Gene ontology analysis of the selected genes shown in (a). (c) Pathway analysis of the selected genes in (a).
